# The invasive Asian bush mosquito *Aedes japonicus* found in the Netherlands can experimentally transmit Zika virus and Usutu virus

**DOI:** 10.1371/journal.pntd.0008217

**Published:** 2020-04-13

**Authors:** Sandra R. Abbo, Tessa M. Visser, Haidong Wang, Giel P. Göertz, Jelke J. Fros, Marleen H. C. Abma-Henkens, Corinne Geertsema, Chantal B. F. Vogels, Marion P. G. Koopmans, Chantal B. E. M. Reusken, Sonja Hall-Mendelin, Roy A. Hall, Monique M. van Oers, Constantianus J. M. Koenraadt, Gorben P. Pijlman

**Affiliations:** 1 Laboratory of Virology, Wageningen University & Research, Wageningen, the Netherlands; 2 Laboratory of Entomology, Wageningen University & Research, Wageningen, the Netherlands; 3 Department of Viroscience, Erasmus Medical Center, Rotterdam, the Netherlands; 4 Public Health Virology, Forensic and Scientific Services, Department of Health, Coopers Plains, Australia; 5 School of Chemistry and Molecular Biosciences, The University of Queensland, St. Lucia, Australia; Institut Pasteur, FRANCE

## Abstract

**Background:**

The Asian bush mosquito *Aedes japonicus* is invading Europe and was first discovered in Lelystad, the Netherlands in 2013, where it has established a permanent population. In this study, we investigated the vector competence of *Ae*. *japonicus* from the Netherlands for the emerging Zika virus (ZIKV) and zoonotic Usutu virus (USUV). ZIKV causes severe congenital microcephaly and Guillain-Barré syndrome in humans. USUV is closely related to West Nile virus, has recently spread throughout Europe and is causing mass mortality of birds. USUV infection in humans can result in clinical manifestations ranging from mild disease to severe neurological impairments.

**Methodology/Principal findings:**

In our study, field-collected *Ae*. *japonicus* females received an infectious blood meal with ZIKV or USUV by droplet feeding. After 14 days at 28°C, 3% of the ZIKV-blood fed mosquitoes and 13% of the USUV-blood fed mosquitoes showed virus-positive saliva, indicating that *Ae*. *japonicus* can transmit both viruses. To investigate the effect of the mosquito midgut barrier on virus transmission, female mosquitoes were intrathoracically injected with ZIKV or USUV. Of the injected mosquitoes, 96% (ZIKV) and 88% (USUV) showed virus-positive saliva after 14 days at 28°C. This indicates that ZIKV and USUV can efficiently replicate in *Ae*. *japonicus* but that a strong midgut barrier is normally restricting virus dissemination. Small RNA deep sequencing of orally infected mosquitoes confirmed active replication of ZIKV and USUV, as demonstrated by potent small interfering RNA responses against both viruses. Additionally, *de novo* small RNA assembly revealed the presence of a novel narnavirus in *Ae*. *japonicus*.

**Conclusions/Significance:**

Given that *Ae*. *japonicus* can experimentally transmit arthropod-borne viruses (arboviruses) like ZIKV and USUV and is currently expanding its territories, we should consider this mosquito as a potential vector for arboviral diseases in Europe.

## Author summary

Arthropod-borne viruses (arboviruses) cause a high disease burden in humans and animals. Zika virus (ZIKV) causes microcephaly and Guillain-Barré syndrome in humans, whereas Usutu virus (USUV) induces high mortality in birds and neurological disease in humans. The spread of arboviruses such as ZIKV and USUV is determined by the presence of mosquitoes that can transmit these viruses from one vertebrate host to the next. Here, we investigate the risk of transmission of ZIKV and USUV by the Asian bush mosquito *Aedes japonicus*. This mosquito is invading Europe and is currently present in the Netherlands. We found that field-collected *Ae*. *japonicus* mosquitoes can experimentally transmit ZIKV and USUV. Of the orally infected mosquitoes, 3% (ZIKV) and 13% (USUV) showed virus-positive saliva after 14 days at 28°C. We also found that ZIKV and USUV activated the antiviral RNA interference immune response of *Ae*. *japonicus*. Moreover, a strong barrier in the mosquito midgut restricted virus dissemination, since 96% (ZIKV) and 88% (USUV) of the mosquitoes injected with ZIKV or USUV showed virus-positive saliva. Additionally, we discovered a narnavirus in *Ae*. *japonicus*. Given that *Ae*. *japonicus* can transmit ZIKV and USUV, we should consider this mosquito as a potential vector for arboviral diseases in Europe.

## Introduction

Unexpected infectious disease outbreaks are increasingly common. Many of these are viral diseases transmitted between vertebrate hosts by arthropod vectors such as mosquitoes, ticks and sandflies. The prevalence of these arthropod-borne viruses (arboviruses) is illustrated by numerous recent outbreaks, fuelled by man-made changes to ecological landscapes in which invertebrate vectors and viruses thrive [1]. In this study we investigate the risk of transmission of the pathogenic flaviviruses Zika virus (ZIKV) and Usutu virus (USUV) by the invasive Asian bush mosquito *Aedes japonicus*.

ZIKV is a mosquito-borne pathogen that was first discovered in the Zika forest of Uganda in a captive, sentinel rhesus monkey in 1947 and in *Aedes africanus* mosquitoes in 1948. Since then, it spread via Asia and the Pacific islands to densely populated regions in the Americas [1,2]. Historically, ZIKV was considered to cause only mild disease with headache, fever, rash, joint pain and muscle pain. However, after its introduction in South America and the start of a large outbreak in humans in 2015, the virus generated worldwide attention due to its severe clinical symptoms, fast spread, and long-term persistence. ZIKV infections in humans caused unexpectedly severe diseases including congenital microcephaly and Guillain-Barré syndrome [3]. ZIKV quickly spread across Central and South America, where the virus established urban transmission cycles involving humans and mosquitoes. The main mosquito vector for ZIKV is the yellow fever mosquito *Aedes aegypti*, but ZIKV transmission by other *Aedes* species, including the Asian tiger mosquito *Aedes albopictus*, has also been reported [4].

USUV was first isolated from mosquitoes in South Africa in 1959 [5], and emerged on the European continent in Italy in 1996 [6]. The first European USUV outbreak became apparent in Austria in 2001 after a sudden mass mortality of birds [5]. Since then, widespread USUV outbreaks in birds have been reported in many European countries, including the Netherlands in 2016 [7]. USUV is primarily transmitted between birds and mosquitoes. The common house mosquito *Culex pipiens* is thought to be an important vector for USUV in Europe [8,9]. In the past years, an increasing number of clinical human USUV infections has been reported while the presence of USUV RNA in donations from healthy blood donors raised concerns for blood safety [10,11]. Symptoms of USUV infection in humans include fever and rash. In addition, USUV has also been linked to human cases of encephalitis [11], underlining the need for awareness of this virus.

The emergence and spread of arboviruses such as ZIKV and USUV are determined by the presence of mosquitoes that can transmit these viruses from one vertebrate host to the next. *Ae*. *japonicus*, belonging to the same genus as the ZIKV vector *Ae*. *aegypti*, has recently received attention due to its potential role in arbovirus transmission [12,13]. This mosquito can transmit multiple flaviviruses of the *Flaviviridae* family including West Nile virus (WNV), Japanese encephalitis virus (JEV), dengue virus and Saint Louis encephalitis virus, but also viruses from other virus families including eastern equine encephalitis virus and chikungunya virus (family *Togaviridae*, genus *Alphavirus*), and La Crosse virus (family *Peribunyaviridae*, genus *Orthobunyavirus*) [12].

*Ae*. *japonicus* is native to Korea, Japan and southern China, and was first detected outside this area in the 1990s. Multiple incursions occurred in New Zealand, however the first established populations outside the native area were reported in eastern states of the USA [12]. *Ae*. *japonicus* is currently present in more than 30 states of the USA and, since 2001, also in Canada. In 2000, *Ae*. *japonicus* was detected for the first time in France, where it was eradicated shortly thereafter [12]. Since this first interception in Europe, permanent *Ae*. *japonicus* populations have been reported in eleven European countries [14]. In the Netherlands, this mosquito was first discovered in 2013, when a female mosquito collected during routine mosquito surveillance in the municipality of Lelystad in 2012, was morphologically and genetically identified as *Ae*. *japonicus*. After extensive surveillance in Lelystad in 2013, the presence of a large *Ae*. *japonicus* population was confirmed [15]. In 2015, a mosquito control program was implemented in Lelystad in an attempt to reduce the population size [16].

*Ae*. *japonicus* is a container-dwelling mosquito, which can colonize diverse natural and man-made habitats. It is an opportunistic feeder; the mosquito feeds on both avian and mammalian hosts, including humans, making it a potential bridge vector for bird-borne zoonotic arboviruses. Importantly, *Ae*. *japonicus* is tolerant to relatively low temperatures, which allows it to successfully expand to regions with a temperate climate [12].

Considering the invasive nature of *Ae*. *japonicus* and its ability to transmit many different arboviruses [12], it is of prime importance to study the potential role of this mosquito in the transmission cycles of newly emerging arboviruses such as ZIKV and USUV. In this study, we determined the vector competence of *Ae*. *japonicus* for ZIKV and USUV. We show that field-collected *Ae*. *japonicus* mosquitoes from the Netherlands can experimentally transmit ZIKV and USUV, and therefore, that *Ae*. *japonicus* could be a potential vector for these arboviruses in Europe. We also found that ZIKV and USUV can efficiently replicate in *Ae*. *japonicus*, but that a mosquito midgut barrier is limiting ZIKV and USUV dissemination. To investigate the effect of mosquito immune responses on virus replication, we studied RNA interference (RNAi) responses against ZIKV and USUV in *Ae*. *japonicus*. The detection of ZIKV- and USUV-derived small interfering RNAs (siRNAs), which are 21 nucleotide (nt) sized RNA products from viral double-stranded RNA (dsRNA) cleavage by the endoribonuclease Dicer-2 [17,18], confirmed active replication of both viruses in *Ae*. *japonicus*. We also investigated whether natural virus infections were present in *Ae*. *japonicus* since these infections could potentially interfere with the vector competence studies. Using *de novo* small RNA assembly, we discovered a novel narnavirus (family *Narnaviridae*; genus *Narnavirus*) in *Ae*. *japonicus*.

## Methods

### Mosquito collection and rearing

**Fig 1:**
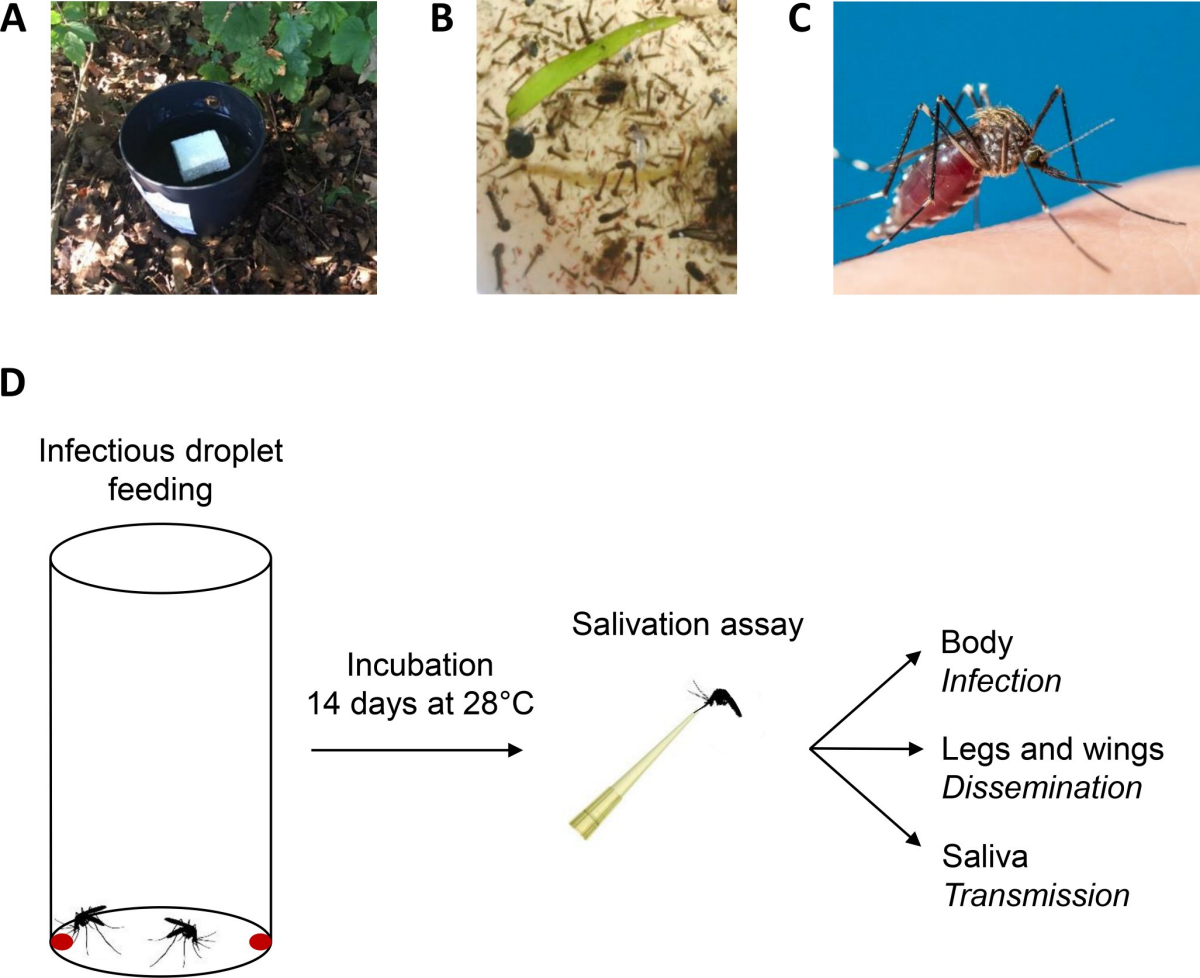
*Ae*. *japonicus* mosquito collection and the infectious droplet feeding experiment. (A) Mosquito eggs were collected using oviposition traps. (B) Mosquito larvae were collected from local rain barrels. (C) An adult female mosquito, which was captured during human landing catches. (D) Infectious droplet feeding and subsequent salivation assays were performed to determine the vector competence of *Ae*. *japonicus* females for ZIKV and USUV.

*Ae*. *japonicus* eggs, larvae and adults were collected in Lelystad, the Netherlands (52°31'42.6"N, 5°28'00.6"E), during August 2017 and July, August and September 2018. Mosquitoes were collected on public and private land. Private land was only accessed when permission was given by the owners to conduct the work on their land. Eggs were collected with oviposition traps (Fig 1A). Each trap consisted of a black plastic flower pot (Elho, Tilburg, the Netherlands) which contained approximately 3.5 litre tap water, hay, and a floating Styrofoam block (length: 6 cm, width: 6 cm, height: 1.5 cm). The Styrofoam blocks were collected and replaced every two weeks. Eggs were found on the sides of the Styrofoam blocks, just above the water surface. Larvae were collected from aboveground water reservoirs in local rain barrels using nets (Fig 1B). Adult females (Fig 1C) were collected by performing human landing catches. During human landing catches, the collector him-/herself acted as bait for mosquitoes. The collector waited for a mosquito to land on him-/herself, and before the mosquito could bite, the mosquito was captured with the use of a mouth aspirator. During the periods of human landing catches in Lelystad, there were no notifications of arbovirus circulation in the area.

The collected *Ae*. *japonicus* larvae and eggs were reared in the laboratory at 26°C, 12:12 light:dark period and 70% relative humidity. Eggs on Styrofoam blocks were hatched in tap water with Liquifry No. 1 (Interpet Ltd., Dorking, UK). A maximum number of 100 eggs was added per plastic tray containing 1.5 litre tap water. Larvae were fed with Tetramin baby fish food (Tetra, Melle, Germany) every 2–3 days. Adults were kept in Bugdorm cages (30 x 30 x 30 cm; MegaView Science Co., Ltd., Taichung, Taiwan) and received a 6% glucose solution as a food source. Adult mosquitoes obtained with human landing catches and adults reared from collected larvae and eggs were pooled and used for experiments. Of the adult mosquitoes used for experiments, 60% originated from field-collected eggs, 35% originated from field-collected larvae and 5% was captured by human landing catches.

*Ae*. *aegypti* mosquitoes (positive control; Rockefeller strain, obtained from Bayer AG, Monheim, Germany) were reared at 27°C with 12:12 light:dark period and 70% relative humidity. Adults were kept in Bugdorm cages (MegaView Science Co., Ltd.) and were provided with 6% glucose solution as a food source. The colony was supplied with human whole blood (from 10 ml tubes coated with lithium heparin; Sanquin Blood Supply Foundation, Nijmegen, the Netherlands) through Parafilm (Heathrow Scientific, Vernon Hills, IL, USA) by the Hemotek PS5 feeder (Discovery Workshops, Lancashire, United Kingdom).

### Cells and viruses

African green monkey kidney Vero E6 cells were cultured as a monolayer in Dulbecco’s Modified Eagle Medium (DMEM; Gibco, Carlsbad, CA, USA) supplemented with 10% fetal bovine serum (FBS; Gibco), penicillin (100 U/ml; Sigma-Aldrich, Saint Louis, MO, USA) and streptomycin (100 μg/ml; Sigma-Aldrich) (P/S). Cells were cultured at 37°C and 5% CO_2_. Prior to virus infections, Vero cells were seeded in HEPES-buffered DMEM medium (Gibco) supplemented with 10% FBS and P/S. When mosquito lysate or saliva was added to the cells, the HEPES-buffered DMEM growth medium was also supplemented with fungizone (2.5 μg/ml of amphotericin B and 2.1 μg/ml of sodium deoxycholate; Gibco) and gentamycin (50 μg/ml; Gibco). This medium is hereafter named DMEM HEPES complete.

Asian tiger mosquito (*Ae*. *albopictus)* C6/36 cells were cultured as a monolayer in Leibovitz L-15 medium (Gibco) supplemented with 10% FBS, 1% nonessential amino acids (Gibco) and 2% tryptose phosphate broth (Gibco). Cells were cultured at 27°C. When mosquito lysate was added to the cells, the Leibovitz L-15 medium was also supplemented with P/S, fungizone (2.5 μg/ml of amphotericin B and 2.1 μg/ml of sodium deoxycholate) and gentamycin (50 μg/ml). This medium is hereafter named Leibovitz L-15 complete.

All procedures involving infectious virus were executed in the biosafety level 3 facility of Wageningen University & Research. Passage 5 and 6 virus stocks of ZIKV, Suriname 2016 (GenBank accession no. KU937936.1; EVAg Ref-SKU 011V-01621; obtained from Erasmus Medical Center, Rotterdam, the Netherlands), and passage 6 virus stocks of USUV, the Netherlands 2016 (GenBank accession no. MH891847.1; EVAg Ref-SKU 011V-02153; obtained from Erasmus Medical Center), were grown on Vero cells. Virus titers were determined by end point dilution assays (EPDAs) on Vero cells.

### Infectious droplet feeding

*Ae*. *japonicus* and *Ae*. *aegypti* females received an infectious blood meal with either ZIKV or USUV by droplet feeding (Fig 1D) as previously described [19]. Groups of 20 mosquitoes were transferred to plastic vials with squeeze foam caps (height of vial: 10 cm, diameter of vial: 5 cm; Carl Roth, Karlsruhe, Germany) and starved for one day. The next day, the mosquitoes were orally infected by feeding on infectious blood droplets. Blood meals were prepared by mixing human blood (Sanquin Blood Supply Foundation), 10% FBS and 1.6% fructose with virus stock to a final virus titer of 1.6 x 10^7^ 50% tissue culture infective dose per millilitre (TCID_50_/ml). Viral titers in the blood meal were afterwards verified by EPDA. For droplet feeding, two 50 μl droplets were provided at the bottom of each plastic vial. Mosquitoes were fed at room temperature with the lights on. After 3–4 hours, the mosquitoes were anesthetized with 100% CO_2_ (continuous supply) and the fully engorged females were selected. These mosquitoes were maintained in netted buckets at 28°C and 12:12 light:dark period for 14 days. A 6% glucose solution was provided as a food source.

### Intrathoracic injection

*Ae*. *japonicus* females received a 69 nl ZIKV injection of 4.4 x 10^3^ TCID_50_ (mosquitoes collected in 2018) or 1.0 x 10^4^ TCID_50_ (mosquitoes collected in 2017), or a 69 nl USUV injection of 3.5 x 10^3^ TCID_50_ in the thorax. *Ae*. *aegypti* females were injected with a 69 nl ZIKV injection of 1.0 x 10^4^ TCID_50_. Mosquitoes were immobilised with 100% CO_2_, and injected using a Drummond Nanoject II Auto-Nanoliter Injector (Drummond Scientific, Broomall, PA, USA) with a glass needle. Injected mosquitoes were kept at 28°C and 12:12 light:dark period for 14 days, and received 6% glucose solution as a food source.

### Salivation assay

Fourteen days post infection, mosquitoes were anesthetized using 100% CO_2_. The legs and wings of each mosquito were removed, collected and stored at -80°C in 1.5 ml SafeSeal micro tubes (Sarstedt, Nümbrecht, Germany) containing 0.5 mm zirconium oxide beads (Next Advance, Averill Park, NY, USA). The mosquito proboscis was inserted into a 200 μl pipet tip containing 5 μl of a 50% FBS and 25% sugar solution in tap water for 45 min to collect mosquito saliva. Afterwards, the mosquito bodies were stored at -80°C in individual 1.5 ml SafeSeal micro tubes (Sarstedt) containing 0.5 mm zirconium oxide beads (Next Advance). Individual mosquito saliva samples were resuspended in 55 μl DMEM HEPES complete and stored at -80°C.

### Infectivity assay

Frozen mosquito body samples and samples containing legs and wings were homogenized in a Bullet Blender Storm (Next Advance) at maximum speed for 2 min. The homogenates were centrifuged in an Eppendorf 5424 centrifuge at 14,500 rpm for 1 min. Afterwards, 100 μl of DMEM HEPES complete was added to each body sample, and 60 μl of DMEM HEPES complete to each legs and wings sample. The homogenates in medium were blended again at maximum speed for 2 min, and centrifuged at 14,500 rpm for 2 min. Thirty μl of each mosquito sample (body, legs and wings, saliva) was added to one well of a 96-well plate containing a monolayer of Vero cells in DMEM HEPES complete. After 2 hours incubation at 37°C, the medium of the cells was replaced by 100 μl fresh DMEM HEPES complete. At 6 days post infection, the wells were scored virus-positive or negative based on cytopathic effect (CPE). The number of virus-positive bodies, legs and wings, or salivas was expressed as a percentage of the total number of analysed mosquitoes.

### Virus titration

Virus titers in TCID_50_/ml were determined by EPDA on Vero cells. This assay measured the virus dilution at which the virus caused CPE in 50% of the inoculated cell cultures. Based on this information, the TCID_50_/ml could then be calculated [20]. Serial tenfold dilutions (10^−1^ till 10^−9^) of virus were made in DMEM HEPES complete. Detached Vero cells were diluted to 5 x 10^5^ cells/ml and added in a 1:1 ratio to the virus dilutions. Of each suspension with diluted virus and Vero cells, 10 μl was added to six wells of a 60-well MicroWell plate (Nunc, Roskilde, Denmark). After 6 days, wells were scored virus-positive or negative based on CPE.

### Immunofluorescence assay

Vero cells were fixed with 4% paraformaldehyde in PBS for 1–4 hours. Cells were washed three times with PBS, permeabilised by incubation in PBS with 0.1% SDS for 10 min, and washed again three times with PBS. Cells were stained with pan-flavivirus α-E (4G2 [21]; mouse monoclonal; dilution 1:50) in PBS with 5% FBS at room temperature for 1 hour. Monolayers were then washed three times with PBS, and stained with goat-α-mouse-Alexa Fluor 488 (dilution 1:2000; Invitrogen, Carlsbad, CA, USA) at 37°C for 1 hour. Cells were washed again three times with PBS and visualised using an Axio Observer Z1m inverted microscope (Zeiss, Jena, Germany) with an X-Cite 120 series lamp.

### Detection of viral RNA replicative intermediates

Pools of approximately ten *Ae*. *japonicus* mosquitoes which were not engorged after a blood meal with ZIKV and incubated at 25°C for 14–29 days, were frozen at -80°C. To prevent potential interference of input virus from the blood meal with the subsequent analysis, mosquitoes were processed at least 14 days post bloodmeal. A total of 28 frozen mosquito pools were blended with 0.5 mm zirconium oxide beads (Next Advance) using a Bullet Blender Storm (Next Advance) at maximum speed for 2 min, and afterwards centrifuged in an Eppendorf 5424 centrifuge at 14,500 rpm for 1 min. One ml of Leibovitz L-15 complete was added to each homogenate. Homogenates were blended again at maximum speed for 2 min, and afterwards centrifuged at 14,500 rpm for 2 min. Next, homogenates were filtered through a 0.2 μm filter (VWR International, Radnor, PA, USA), and collected into fresh tubes. Fifty μl of filtered homogenate was inoculated onto four wells with C6/36 cells in a 96-well plate. After incubation at 27°C for 7 days, the presence of viral RNA replicative intermediates was tested by enzyme-linked immunosorbent assay using monoclonal antibodies to viral dsRNA intermediates in cells (MAVRIC) [22]. The newly discovered insect-specific flavivirus Binjari virus [23] was used as a positive control. Briefly, medium was removed and cells were fixed and permeabilised on ice for 10 min using 4% paraformaldehyde and 0.5% Triton X in PBS. After removal of the fixative, cells were dried overnight. The next day, cells were blocked with 1% milk powder in PBS with 0.05% TWEEN 20 (PBS-T) at room temperature for 40 min. Monolayers were stained with α-dsRNA (3G1.1 [22]; mouse monoclonal; dilution 1:32) in blocking solution at 37°C for 1 hour. Cells were washed four times with PBS-T, and stained with goat-α-mouse-HRP (dilution 1:2000; Dako, Santa Clara, CA, USA) in blocking solution at 37°C for 1 hour. Afterwards, monolayers were washed six times with PBS-T. To prepare the substrate buffer, 0.2 M Na_2_HPO_4_ was added to 0.1 M citric acid until a pH of 4.2 was reached. 2,2’-azino-bis(3-ethylbenzthiazoline-6-sulfonic acid) (ABTS) and H_2_O_2_ were mixed with the beforementioned buffer to reach final molar concentrations of 1 mM and 3 mM respectively, and this buffer with substrate was added to the cells. Cells were incubated at room temperature in the dark. After 1 hour, absorbance was measured at 405 nm using a FLUOstar OPTIMA microplate reader (BMG LABTECH, Ortenberg, Germany).

### RNA extraction

Total RNA was extracted from cells using TRIzol reagent (Invitrogen) according to manufacturer’s instructions. For RNA isolation from mosquito bodies, the bodies were first blended in the Bullet Blender Storm (Next Advance) using 0.5 mm zirconium oxide beads (Next Advance) at maximum speed for 2 min, and afterwards centrifuged in an Eppendorf 5424 centrifuge at 14,500 rpm for 1 min. 100 μl of DMEM HEPES complete was added to each homogenate. Homogenates were again blended at maximum speed for 2 min. After centrifugation at 14,500 rpm for 2 min, the medium was removed and used for infectivity assays. 1 ml of TRIzol reagent was added to the pellet. Pools of 4–6 mosquito homogenates were collected in 1 ml TRIzol reagent. Mosquito total RNA was isolated as described above. An additional 75% ethanol wash was included. RNA yields were determined using a NanoDrop ND-1000 spectrophotometer.

### Reverse transcriptase PCR

Reverse transcriptase PCR (RT-PCR) was performed with 100 ng total RNA per reaction using a 2720 Thermal Cycler (Applied Biosystems, Foster City, CA, USA) and the SuperScript III One-Step RT-PCR System with Platinum *Taq* DNA polymerase (Invitrogen) according to manufacturer’s instructions. Primers targeting the region coding for ZIKV non-structural protein 1 (NS1) (forward: 5’-GAGACGAGATGCGGTACAGG-3’; reverse: 5’-CGACCGTCAGTTGAACTCCA-3’) and the region encoding USUV non-structural protein 5 (NS5) (forward: 5’-GGCTGTAGAGGACCCTCGG-3’; reverse: 5’-GACTGCCTTTCGCTTTGCCA-3’) were used at annealing temperatures of 55°C and 60°C, respectively.

### Small RNA deep sequencing

Total RNA was extracted from two pools of *Ae*. *japonicus* mosquitoes that were found virus-positive after blood droplet feeding with either ZIKV or USUV. The pool with ZIKV-infected mosquitoes consisted of six mosquitoes, whereas the pool with USUV-infected mosquitoes contained four mosquitoes. Twenty μl of mosquito total RNA with a concentration of 250 ng/μl was sent to BGI (‎Shenzhen, Guangdong, China) for small RNA sequencing as previously described [24]. Single-end FASTQ reads were generated with an in-house filtering protocol of BGI. Small RNA sequencing libraries have been uploaded to the NCBI sequence read archive (SRA) under BioProject PRJNA545039.

### Small RNA analysis

Small RNA analysis was performed using the Galaxy webserver [25]. Small RNA sequences were mapped with Bowtie 2 [26] version 2.3.4.2 allowing 1 mismatch and a seed length of 28. Reads were mapped against the ZIKV, Suriname 2016 genome (GenBank accession no. KU937936.1) or the USUV, the Netherlands 2016 genome (GenBank accession no. MH891847.1). Since the Netherlands 2016 USUV sequence did not have complete sequences of the untranslated regions (UTRs), it was complemented with the 5’ UTR sequence of a closely related USUV isolate from the Netherlands (GenBank accession no. KY128482.1) and the 3’ UTR sequence of USUV, Italy 2012 (GenBank accession no. KX816650.1). Next, size distribution profiles of the viral small RNAs were made from all mapped reads. The read counts of the size distribution profiles were normalized as percentages of the total number of reads in the library. The 5’-ends of 21 nt sized virus reads were mapped to the viral genome to generate siRNA genome distributions. The number of 21 nt reads per location on the genome was calculated as a percentage of the total number of reads in the library. The 25–30 nt small RNAs derived from ZIKV or USUV were analysed for PIWI-interacting RNA (piRNA) signatures using Weblogo3. Sequence overlaps between the putative piRNAs were analysed using the small RNA signatures tool [27] version 3.1.0 on the mississippi.snv.jussieu.fr Galaxy server using 25–30 nt small RNAs as input. *De novo* assembly from small RNA reads was done as previously described [24].

## Results

### Collection of *Ae*. *japonicus* from Lelystad, the Netherlands in the summers of 2017 and 2018

*Ae*. *japonicus* mosquitoes were collected from Lelystad, the Netherlands in the summers of 2017 and 2018. In total, 3,130 mosquito eggs were collected in 2017 and 5,435 eggs in 2018. Human landing catches during two days in July 2018 resulted in a total of 17 captured females. In addition, over 60 *Ae*. *japonicus* females were collected during two days in August and September 2018. These findings confirm that *Ae*. *japonicus* mosquitoes from Lelystad have an interest to feed on human hosts.

### *Ae*. *japonicus* from the Netherlands can experimentally transmit ZIKV and USUV

To investigate the vector competence of *Ae*. *japonicus* for ZIKV and USUV, female mosquitoes received a blood meal with either ZIKV or USUV by droplet feeding (Fig 1D). As a positive control, ZIKV-competent *Ae*. *aegypti* mosquitoes [28] were also offered an infectious blood meal containing ZIKV by droplet feeding. This particular method was used because *Ae*. *japonicus* did not feed on blood meals offered via the Hemotek system, with either a Parafilm membrane or a pig intestine membrane. The percentage of *Ae*. *japonicus* females that took up a blood meal varied from 1–33% depending on the experiment, whereas the percentage of *Ae*. *aegypti* females that engorged a blood meal during droplet feeding was low with feeding percentages of maximum 5%. After 14 days, the presence of virus in the mosquito body, legs and wings, and saliva was determined by infectivity assays on Vero cells. For a subset of the results, these scores based on CPE of Vero cells were confirmed by RT-PCR using primers targeting NS1 (ZIKV) or NS5 (USUV).

**Fig 2:**
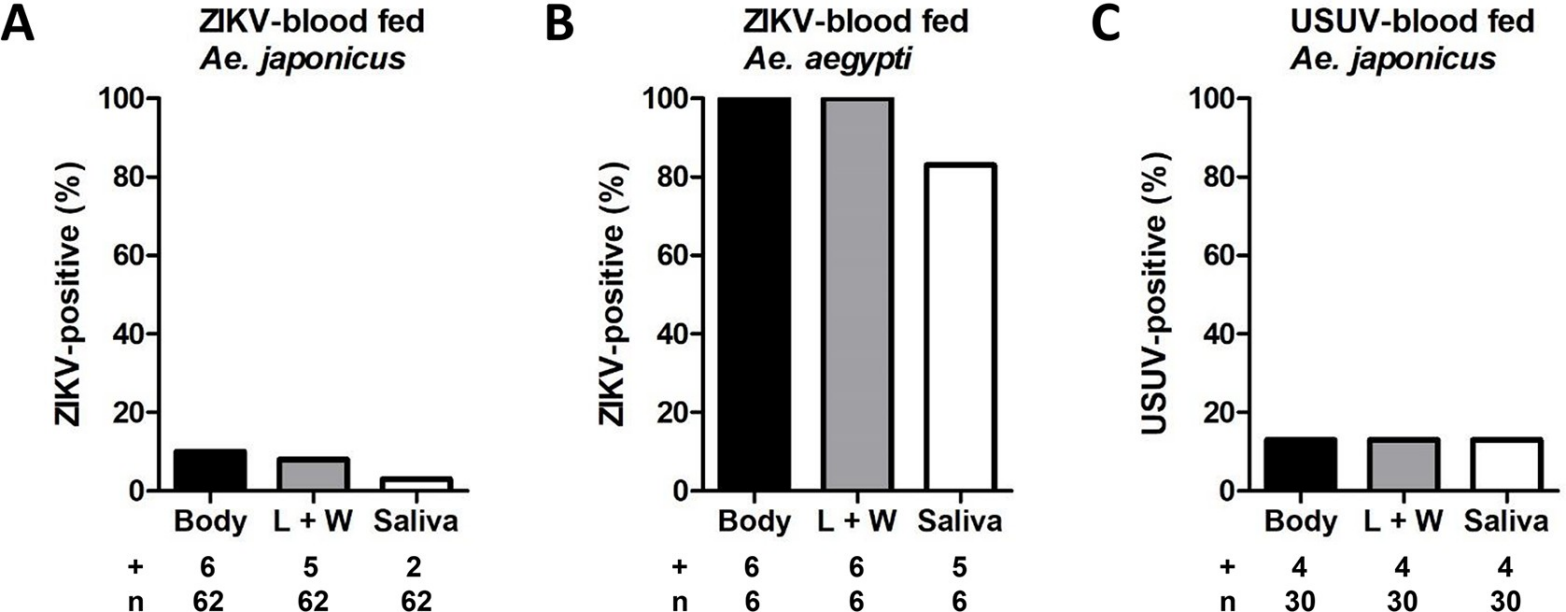
*Ae*. *japonicus* can experimentally transmit ZIKV and USUV. *Ae*. *japonicus* mosquitoes received an infectious blood meal with ZIKV or USUV, and were subsequently incubated at 28°C for 14 days. As a positive control, *Ae*. *aegypti* mosquitoes were offered an infectious blood meal with ZIKV. The percentage of virus-positive bodies, legs and wings (L + W) and salivas out of the total number of mosquitoes tested (number positive (+) / total number tested (n)) was determined for (A) ZIKV-blood fed *Ae*. *japonicus* mosquitoes, (B) ZIKV-blood fed *Ae*. *aegypti* mosquitoes and (C) USUV-blood fed *Ae*. *japonicus* mosquitoes.

The combined results from five (*Ae*. *japonicus* with ZIKV), three (*Ae*. *aegypti* with ZIKV) and three (*Ae*. *japonicus* with USUV) independent experiments are shown in Fig 2. After a blood meal with ZIKV and subsequent incubation at 28°C for 14 days, 10% of the *Ae*. *japonicus* mosquitoes had virus-positive bodies, 8% had virus-positive legs and wings, and 3% had detectable ZIKV in their saliva (Fig 2A). For *Ae*. *aegypti*, 100% of the bodies and legs and wings were ZIKV-positive, and 83% of the mosquitoes showed ZIKV-positive saliva (Fig 2B), suggesting that *Ae*. *aegypti* is a more competent vector for ZIKV compared to *Ae*. *japonicus*. Of the *Ae*. *japonicus* mosquitoes orally exposed to USUV, 13% had virus-positive bodies, legs and wings, and salivas (Fig 2C). These results demonstrate that *Ae*. *japonicus* is able to experimentally transmit ZIKV and USUV.

**Fig 3:**
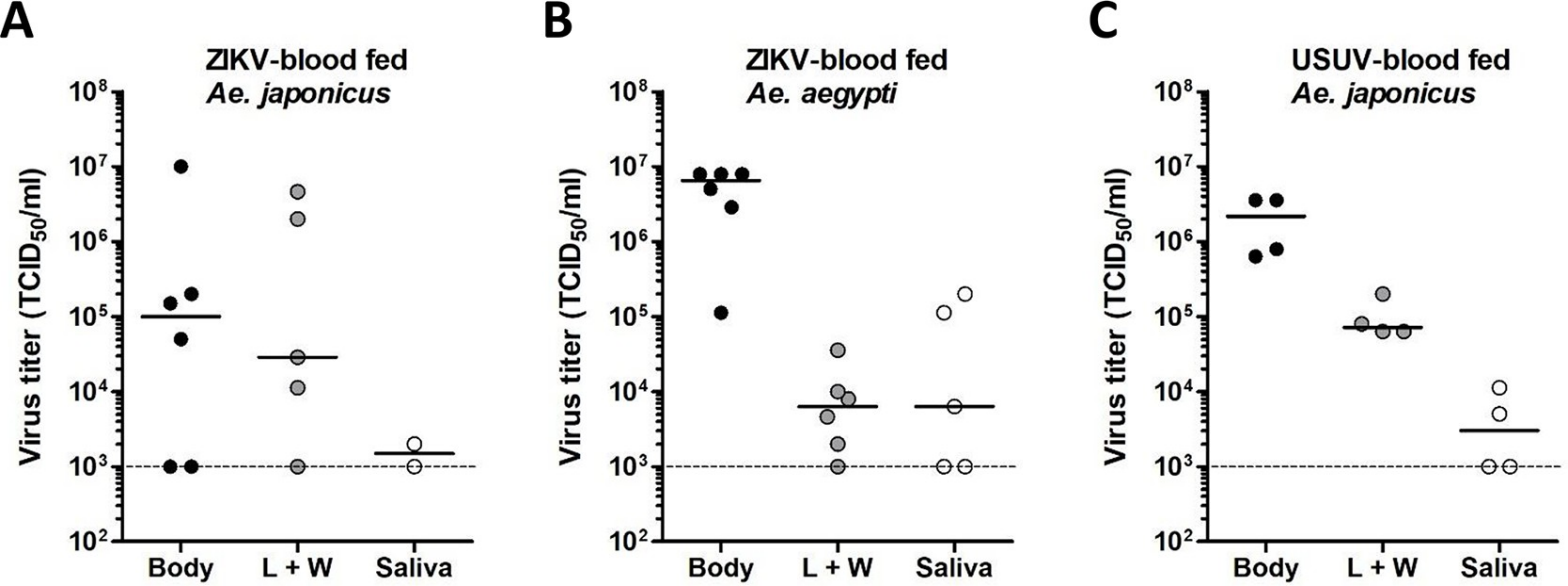
ZIKV and USUV can replicate to high viral titers in *Ae*. *japonicus* after oral infection. Viral titers of the mosquito bodies, legs and wings (L + W), and salivas were determined by EPDA for (A) ZIKV-blood fed *Ae*. *japonicus* mosquitoes, (B) ZIKV-blood fed *Ae*. *aegypti* mosquitoes, and (C) USUV-blood fed *Ae*. *japonicus* mosquitoes. Data points represent individual mosquitoes infected with either ZIKV or USUV. Lines show the median viral titers. Dashed lines show the detection limit of the EPDA.

Viral titers were determined for the ZIKV- and USUV-positive mosquito bodies, legs and wings, and salivas by EPDA (Fig 3). For ZIKV-positive *Ae*. *japonicus* mosquitoes, titers were variable, with median titers of 1.0 x 10^5^ TCID_50_/ml in the body, 2.9 x 10^4^ TCID_50_/ml in the legs and wings, and 1.5 x 10^3^ TCID_50_/ml in the saliva (Fig 3A). Interestingly, the two *Ae*. *japonicus* mosquitoes with ZIKV-positive saliva also showed the highest viral titers in the legs and wings, suggesting that strong virus dissemination is needed for the virus to accumulate in the saliva. ZIKV-positive *Ae*. *aegypti* mosquitoes showed median viral titers of 6.5 x 10^6^ TCID_50_/ml in the body, 6.3 x 10^3^ TCID_50_/ml in the legs and wings, and 6.3 x 10^3^ TCID_50_/ml in the saliva (Fig 3B). USUV-positive *Ae*. *japonicus* mosquitoes showed median viral titers of 2.2 x 10^6^ TCID_50_/ml in the body, 7.1 x 10^4^ TCID_50_/ml in the legs and wings, and 3.0 x 10^3^ TCID_50_/ml in the saliva (Fig 3C). Thus, *Ae*. *japonicus* mosquitoes showing fully disseminated ZIKV and USUV infections and also virus-positive salivas were detected.

### No natural flavivirus infections detected in field-collected *Ae*. *japonicus* mosquitoes

The low ZIKV and USUV transmission reported for *Ae*. *japonicus* after an infectious blood meal could theoretically be due to a natural flavivirus infection already present in the field-collected mosquitoes that is preventing virus transmission of the newly introduced ZIKV and USUV [29–31]. To exclude potential natural flavivirus infections in *Ae*. *japonicus* that could have interfered with the vector competence studies, MAVRIC analysis [22] was performed on a total of 228 *Ae*. *japonicus* females (subdivided over 28 pools). Females that were offered a ZIKV blood meal but did not show engorgement afterwards, were incubated at 25°C for 14–29 days and subsequently analysed using MAVRIC. Out of the 28 pools, 6 were found to be virus-positive, however all of these 6 pools were shown to be RT-PCR positive for ZIKV. This indicated that *Ae*. *japonicus* mosquitoes could become ZIKV-positive even though the mosquitoes were not engorged and must have taken up only a very small volume of infectious blood. We conclude that no natural replicating flaviviruses were present in *Ae*. *japonicus* mosquitoes used in the vector competence assays. Importantly, this experiment also showed that ZIKV can replicate in *Ae*. *japonicus* at an incubation temperature of 25°C.

### A mosquito midgut barrier limits ZIKV and USUV dissemination in *Ae*. *japonicus*

The vector competence experiments indicated that only 3% of the ZIKV-blood fed *Ae*. *japonicus* mosquitoes and 13% of the USUV-blood fed *Ae*. *japonicus* mosquitoes were able to transmit the virus. To investigate whether a mosquito midgut barrier and/or a salivary gland barrier is limiting virus dissemination, *Ae*. *japonicus* mosquitoes were intrathoracically injected with either ZIKV or USUV to bypass the midgut barrier. *Ae*. *japonicus* mosquitoes were injected with 4.4 x 10^3^ TCID_50_ of ZIKV during four independent experiments or 1.0 x 10^4^ TCID_50_ of ZIKV during three independent experiments. As a positive control, *Ae*. *aegypti* mosquitoes were also injected with ZIKV in three independent experiments. Injections of *Ae*. *japonicus* with USUV were done in two independent experiments. The injected mosquitoes were incubated at 28°C for 14 days, and afterwards the mosquito bodies, legs and wings, and salivas were checked for the presence of infectious virus by infectivity assays on Vero cells. The inoculated Vero cells were scored based on CPE, and a subset of the results was also validated using RT-PCR with primers against ZIKV NS1 or USUV NS5. In addition, the findings were also confirmed by immunofluorescence assays using 4G2 panflavivirus α-E.

**Fig 4:**
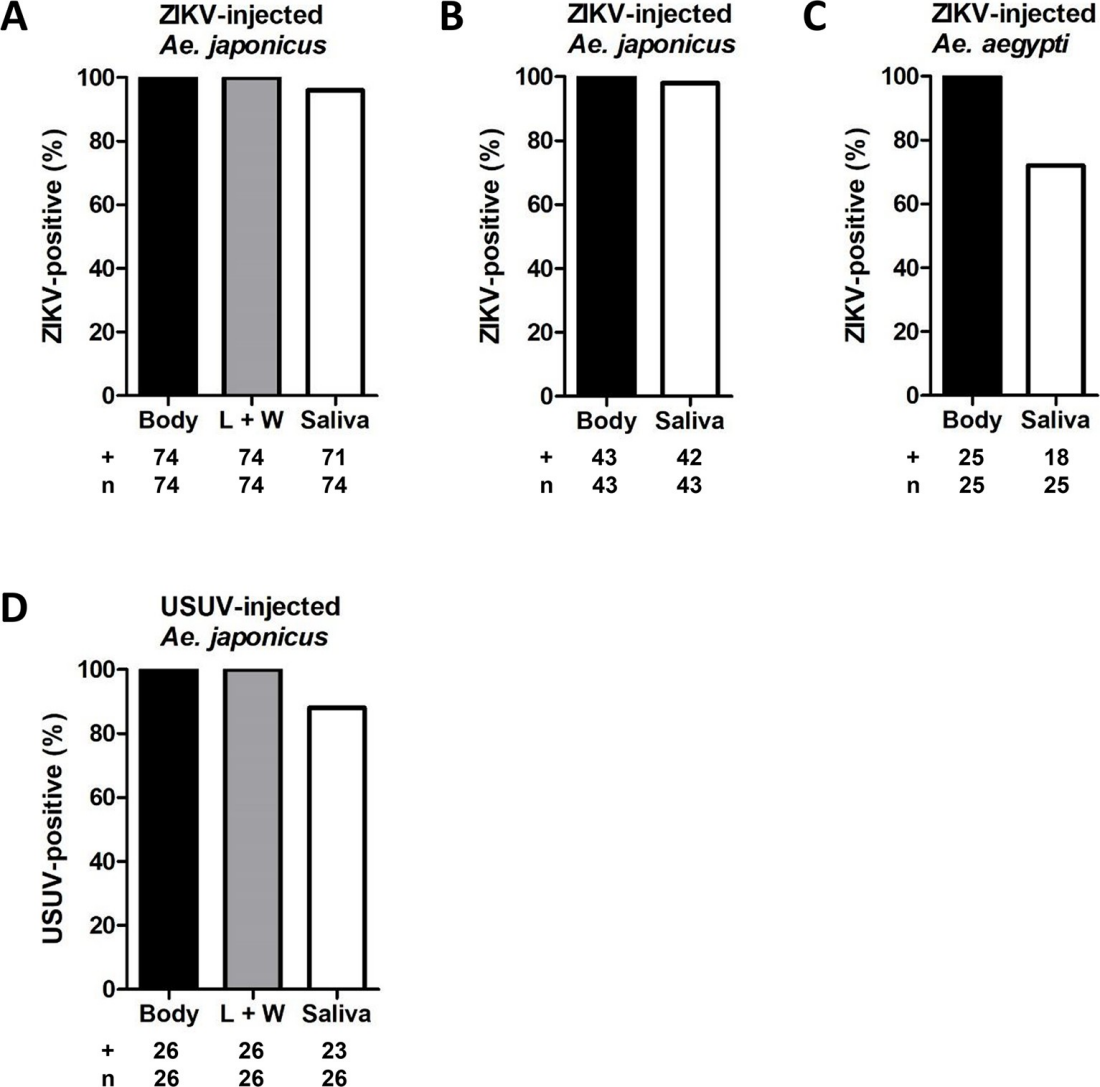
After intrathoracic injection, ZIKV and USUV can fully disseminate in *Ae*. *japonicus*. *Ae*. *japonicus* mosquitoes were intrathoracically injected with ZIKV or USUV. *Ae*. *aegypti* mosquitoes, injected with ZIKV, were included as a positive control. After injection, mosquitoes were incubated at 28°C for 14 days. The percentage of virus-positive bodies, legs and wings (L + W) and salivas out of the total number of mosquitoes tested (number positive (+) / total number tested (n)) was determined for (A) *Ae*. *japonicus* mosquitoes injected with 4.4 x 10^3^ TCID_50_ of ZIKV, (B) *Ae*. *japonicus* mosquitoes injected with 1.0 x 10^4^ TCID_50_ of ZIKV, (C) *Ae*. *aegypti* mosquitoes injected with ZIKV and (D) *Ae*. *japonicus* mosquitoes injected with USUV.

The combined results are shown in Fig 4. Of all injected mosquitoes, 100% showed virus-positive bodies. Moreover, the observed viral dissemination for 100% of the injected *Ae*. *japonicus* mosquitoes indicated that both ZIKV and USUV can replicate in *Ae*. *japonicus* and disseminate to the mosquito legs and wings. Very efficient transmission was observed for ZIKV (96% of the analysed mosquitoes showed ZIKV-positive saliva after an injection with 4.4 x 10^3^ TCID_50_, and 98% after an injection with 1.0 x 10^4^ TCID_50_; Fig 4A and 4B), indicating that there is no salivary gland barrier against ZIKV present in *Ae*. *japonicus*. For *Ae*. *aegypti*, a better salivary gland barrier against ZIKV was observed, as only 72% of the injected mosquitoes showed virus-positive saliva (Fig 4C), which is in agreement with our earlier studies [28]. Additionally, 88% of the USUV-injected *Ae*. *japonicus* showed USUV accumulation in the saliva (Fig 4D), which indicated that USUV, like ZIKV, does not encounter a strong salivary gland barrier in *Ae*. *japonicus*. We thus found that both ZIKV and USUV can efficiently replicate in *Ae*. *japonicus* after intrathoracic injection. However, only a low percentage of the *Ae*. *japonicus* mosquitoes became virus-positive after an infectious blood meal with high titer, which shows the existence of a midgut barrier that strongly limits virus dissemination through the mosquito and lowers virus transmission as a result.

**Fig 5:**
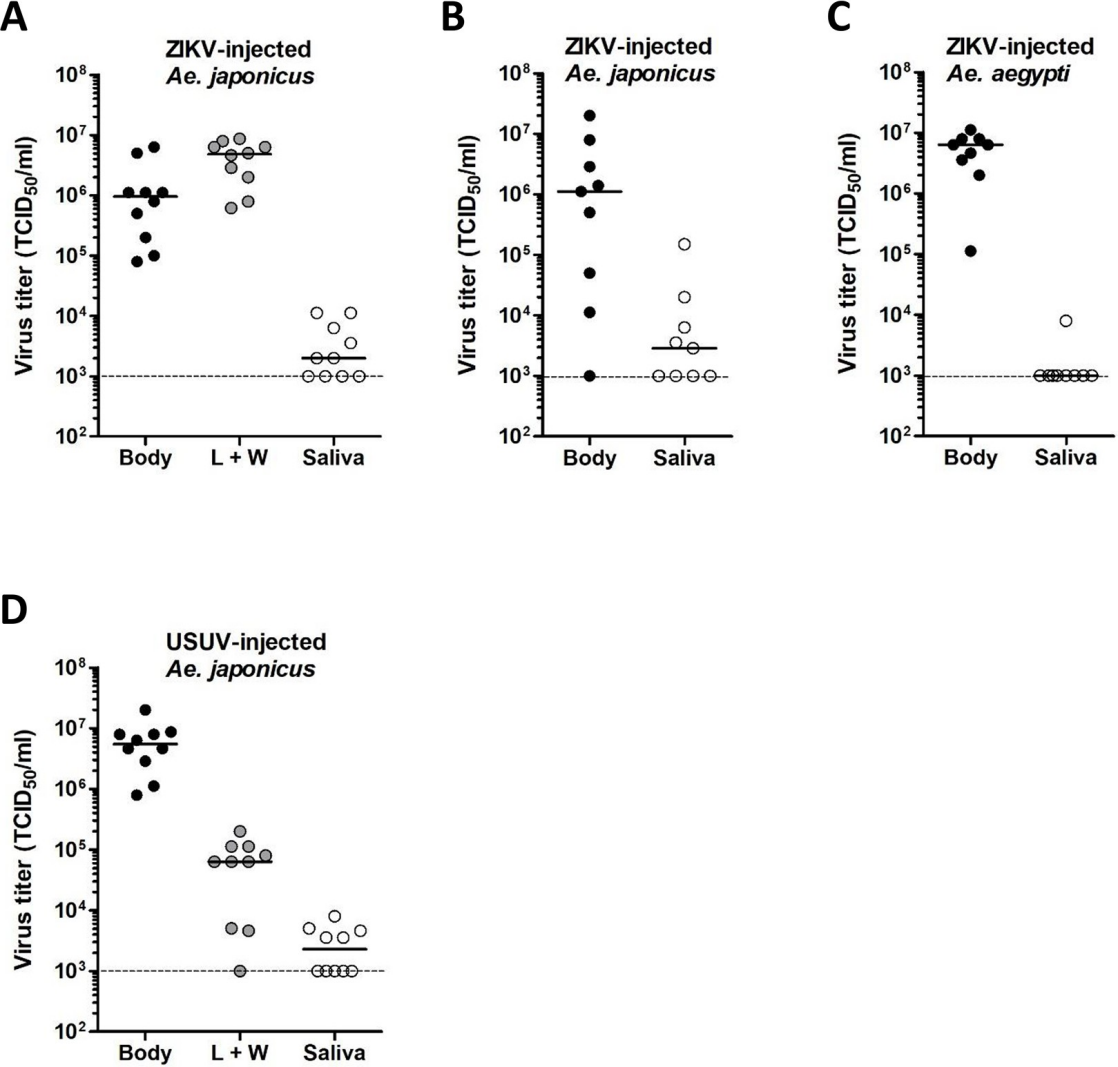
ZIKV and USUV can replicate to high viral titers in *Ae*. *japonicus* after intrathoracic injection. Viral titers of the mosquito bodies, legs and wings (L + W), and salivas were determined by EPDA for (A) *Ae*. *japonicus* mosquitoes injected with 4.4 x 10^3^ TCID_50_ of ZIKV, (B) *Ae*. *japonicus* mosquitoes injected with 1.0 x 10^4^ TCID_50_ of ZIKV, (C) *Ae*. *aegypti* mosquitoes injected with ZIKV and (D) *Ae*. *japonicus* mosquitoes injected with USUV. Data points represent individual mosquitoes intrathoracically injected with either ZIKV or USUV. Lines indicate the median viral titers. Dashed lines show the detection limit of the EPDA.

Viral titers were measured for the ZIKV- and USUV-injected mosquitoes by EPDA (Fig 5). *Ae*. *japonicus* mosquitoes injected with 4.4 x 10^3^ TCID_50_ of ZIKV reached median viral titers of 9.6 x 10^5^ TCID_50_/ml in the body, 4.8 x 10^6^ TCID_50_/ml in the legs and wings, and 2.0 x 10^3^ TCID_50_/ml in the saliva (Fig 5A). *Ae*. *japonicus* mosquitoes injected with 1.0 x 10^4^ TCID_50_ of ZIKV showed median viral titers of 1.1 x 10^6^ TCID_50_/ml in the body, and 2.9 x 10^3^ TCID_50_/ml in the saliva (Fig 5B). The median viral titers for ZIKV-injected *Ae*. *aegypti* mosquitoes were 6.3 x 10^6^ TCID_50_/ml in the body, and below the detection limit of 1.0 x 10^3^ TCID_50_/ml in the saliva (Fig 5C). USUV-injected *Ae*. *japonicus* mosquitoes showed median viral titers of 5.5 x 10^6^ TCID_50_/ml in the body, 6.3 x 10^4^ TCID_50_/ml in the legs and wings, and 2.3 x 10^3^ TCID_50_/ml in the saliva (Fig 5D). The results show that high median viral titers in bodies and legs and wings were found for both ZIKV- and USUV-injected *Ae*. *japonicus* mosquitoes. This indicated that both ZIKV and USUV can efficiently replicate in *Ae*. *japonicus*, which is also supported by the fact that both viruses can easily cross the mosquito salivary gland barrier and induce high viral titers in the saliva.

### ZIKV and USUV induce strong viral siRNA responses in *Ae*. *japonicus*

**Fig 6:**
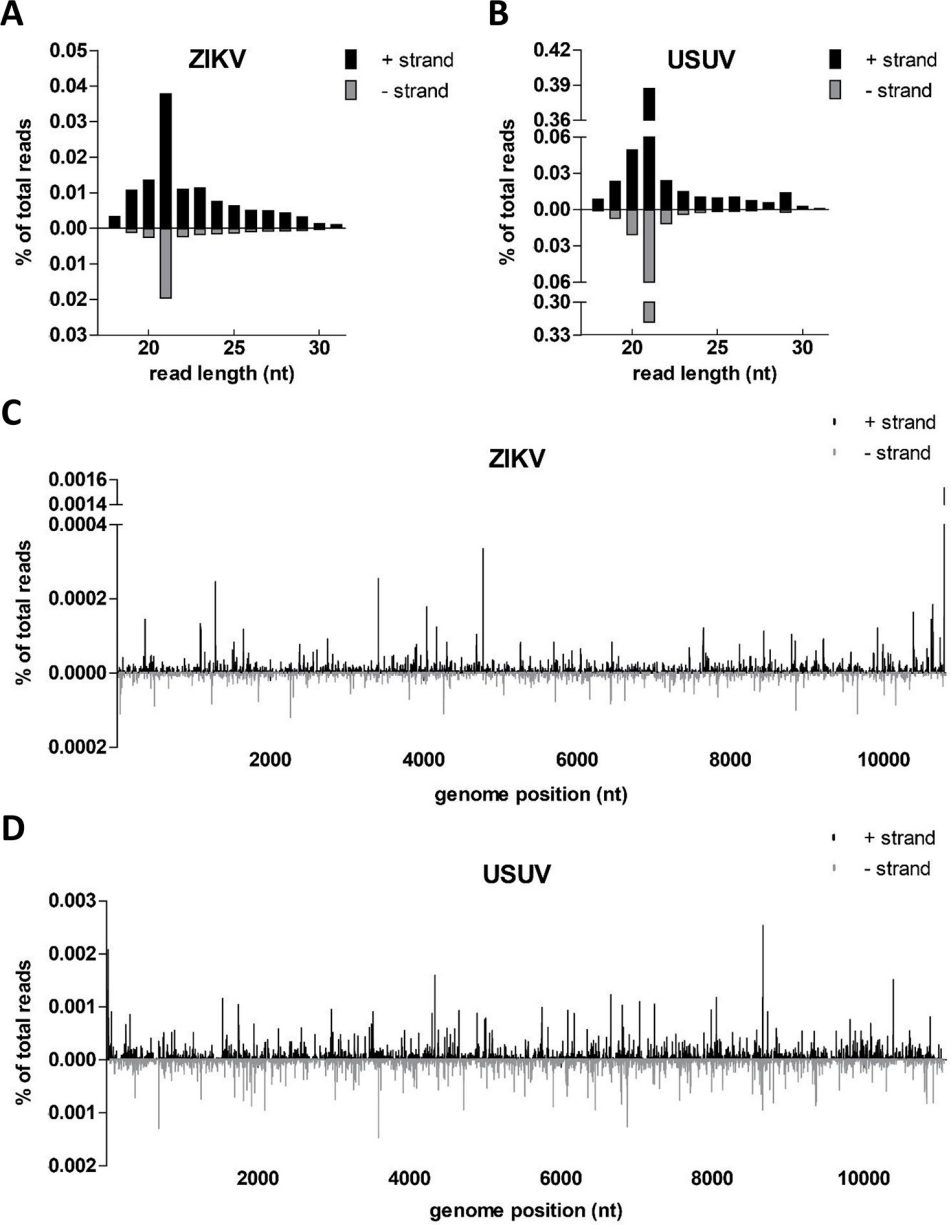
Small RNA sequencing revealed 21 nt siRNA responses against ZIKV and USUV in *Ae*. *japonicus*. The size distribution profiles of small RNAs that mapped to the genomes of (A) ZIKV or (B) USUV are indicated. The distribution of 21 nt (C) ZIKV-derived or (D) USUV-derived siRNAs across the viral genome is shown. Viral reads mapping to the positive-sense viral RNA strand are depicted above the X-axes and presented in black; viral reads mapping to the negative-sense viral RNA strand are depicted below the X-axes and presented in grey. The viral small RNA read counts were normalized against the total library of small RNA reads and are presented as a percentage of the total number of small RNAs in the library.

Viral infection in insects induces antiviral responses, of which RNAi is considered to be an important pathway [17]. During flavivirus replication in mosquitoes, a viral dsRNA intermediate is formed in viral replication complexes, which is recognized and processed by the RNAi machinery. The endoribonuclease Dicer-2 recognizes and cleaves dsRNA into 21 nt siRNAs [17,18]. In addition to viral siRNAs, the production of 25–30 nt viral piRNAs has been reported for arbovirus-infected *Aedes* species [32,33]. To the best of our knowledge, the small RNA responses of *Ae*. *japonicus* against arboviral infections have never been studied. Detailed analysis of the small RNA deep sequencing libraries derived from *Ae*. *japonicus* mosquitoes revealed a high abundance of 21 nt small RNA reads mapping to both the positive-sense and negative-sense viral RNA strands of ZIKV and USUV, which indicates strong small RNA responses against both viruses (Fig 6). This further confirmed active replication of ZIKV and USUV in *Ae*. *japonicus*. USUV-infected mosquitoes showed stronger RNAi responses compared to ZIKV-infected mosquitoes, because a higher percentage of the total number of reads mapped to the USUV genome compared to the percentage of the total number of reads that mapped to the ZIKV genome (Fig 6A and 6B).

For both ZIKV and USUV, 21 nt viral-derived siRNAs were the most abundant group of viral small RNAs (Fig 6A and 6B). A shoulder of 25–30 nt sized viral small RNAs was also observed, which is in the size range of piRNAs. These small RNAs were analysed for the presence of the characteristic piRNA signature (10 nt overlap and 1U/10A sequence bias) caused by the ping-pong piRNA amplification cycle [32,33]. No such signatures were identified. For ZIKV and USUV, 21 nt viral siRNAs mapped across the entire viral RNA genome on both the positive and negative strand (Fig 6C and 6D), with a major hot spot of ZIKV siRNAs at the 3’ stem-loop (SL) of the ZIKV 3’ UTR. Thus, the ZIKV 3’ SL is preferentially processed by the RNAi machinery into 21 nt siRNAs.

### Discovery of a novel narnavirus in *Ae*. *japonicus*

**Fig 7:**
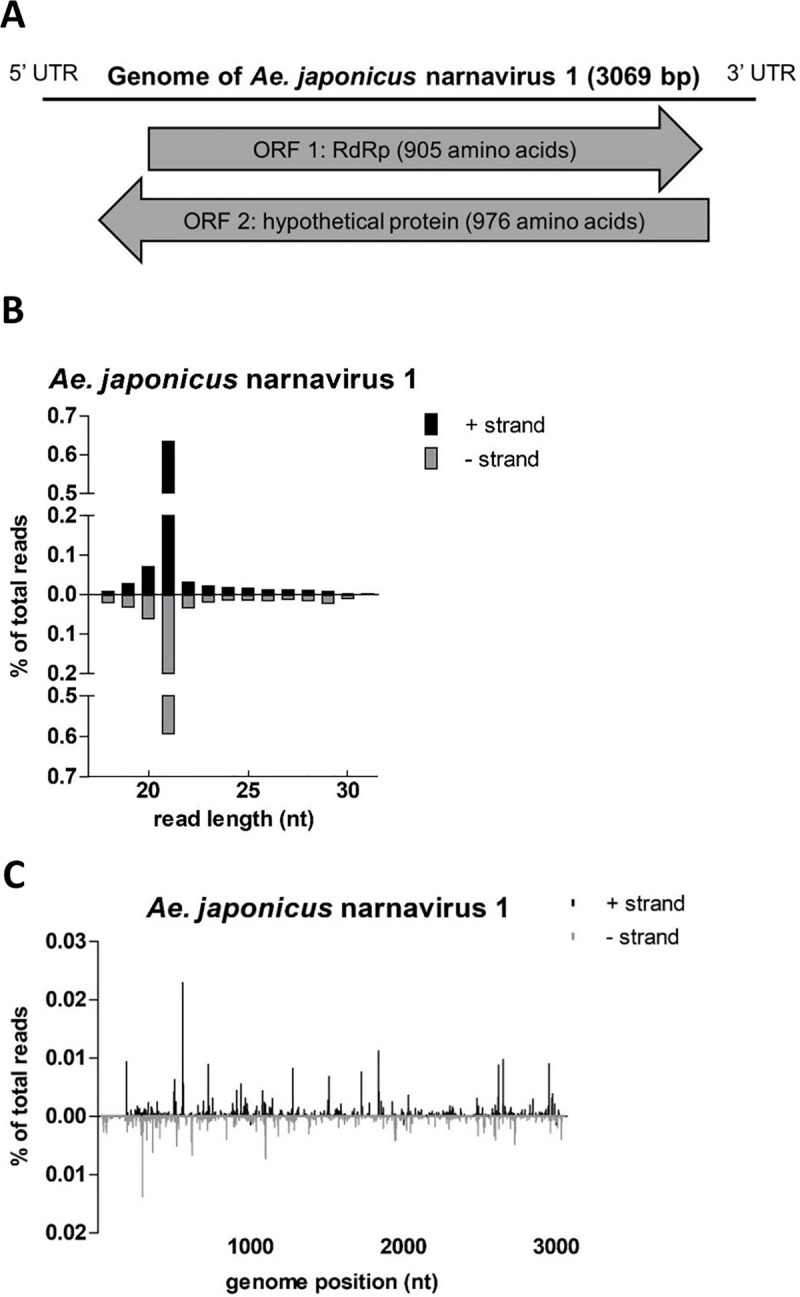
*De novo* small RNA assembly revealed the presence of a narnavirus in *Ae*. *japonicus*. (A) Schematic representation of the predicted coding strategy of AejapNV1. (B) Size distribution profile of small RNAs that mapped to the genome of AejapNV1. (C) Distribution of 21 nt AejapNV1-derived siRNAs across the viral genome. Viral reads mapping to the positive-sense viral RNA strand are depicted above the X-axis and presented in black; viral reads mapping to the negative-sense viral RNA strand are depicted below the X-axis and presented in grey. The viral small RNA read counts were normalized against the total library of small RNA reads and are presented as a percentage of the total number of small RNAs in the library.

*De novo* assembly of small RNA reads revealed the presence of a novel narnavirus (family *Narnaviridae*; genus *Narnavirus*) in *Ae*. *japonicus*. This narnavirus was detected in both pools of *Ae*. *japonicus* mosquitoes which were sent for small RNA sequencing, and was named *Ae*. *japonicus* narnavirus 1 (AejapNV1). The genome sequence of AejapNV1 was uploaded to GenBank (accession no. MK984721). The 3069 base pair (bp) genome of AejapNV1 contains two open reading frames (ORFs) (Fig 7A). The first ORF, present on the positive-sense viral RNA strand, encodes an RNA-dependent RNA polymerase (RdRp). The second ORF, present on the negative-sense viral RNA strand, codes for a hypothetical protein with no known homology. Of the small RNAs induced by AejapNV1, 21 nt siRNAs were most prevalent (Fig 7B) and mapped across the genome (Fig 7C).

## Discussion

*Ae*. *japonicus* is an invasive mosquito species and a potential vector for a panel of arboviruses, including JEV and WNV [12,19,34,35]. Here we show for the first time that field-collected *Ae*. *japonicus* mosquitoes from the Netherlands can experimentally transmit ZIKV and USUV. So far, no ZIKV- or USUV-positive *Ae*. *japonicus* mosquitoes have been found in the Netherlands, but a pool of *Ae*. *japonicus* mosquitoes with disseminated USUV infection has recently been collected from Graz in Austria [36]. This finding could suggest a potential role of *Ae*. *japonicus* in the transmission cycle of USUV in Europe. Currently, there is no evidence of ZIKV transmission by *Ae*. *japonicus* in the field. Nonetheless, 10% of the *Ae*. *japonicus* mosquitoes could transmit ZIKV in a laboratory study with *Ae*. *japonicus* mosquitoes from south-western Germany at 27°C [19], which was slightly higher than the 3% found in our study. Furthermore, the infection rates greatly differed between the German *Ae*. *japonicus* mosquitoes (67%) [19] and the *Ae*. *japonicus* mosquitoes from the Netherlands (10%). This might be explained by technical differences between both studies. In the study with *Ae*. *japonicus* mosquitoes from Germany, the presence of ZIKV in mosquito bodies was determined by quantitative real-time RT-PCR directly on mosquito bodies [19], whereas in our study, mosquito bodies were only considered ZIKV-positive when the inoculation of supernatant from blended bodies on Vero cells resulted in CPE. A real-time RT-PCR assay cannot distinguish viral RNA from infectious virus particles, whereas infectivity assays on Vero cells solely detect infectious virus. The higher infection rate reported for *Ae*. *japonicus* from Germany could therefore be explained by the (perhaps more sensitive) detection of viral RNA. But it could also be that the RT-PCR detects residual input RNA from the blood meal. The percentage of mosquitoes showing virus dissemination out of the total number of mosquitoes tested was similar for *Ae*. *japonicus* from Germany (10%) [19] and the Netherlands (8%), which might indeed indicate equal percentages of mosquito bodies with infectious, replicating virus.

RNAi is considered an essential antiviral defence mechanism in insects, including mosquitoes. Here we show that *Ae*. *japonicus* induces strong small RNA responses against both ZIKV and USUV, with small RNA reads mapping to both the positive and negative viral RNA strands of ZIKV and USUV. RNAi responses against USUV were stronger than the RNAi responses against ZIKV. This could be explained by the fact that the median viral body titer of the analysed USUV-infected mosquitoes was approximately 20-fold higher compared to the median viral body titer of the ZIKV-infected mosquitoes (Fig 3A and 3C). For ZIKV, only two out of six mosquitoes within the analysed pool showed fully disseminated viral infection including virus accumulation in the mosquito saliva, whereas for the USUV-infected mosquitoes, all four analysed mosquitoes had fully disseminated infections including the presence of virus in the saliva. Thus, a better disseminated USUV infection in *Ae*. *japonicus* mosquitoes is associated with a stronger RNAi response, probably because there is a higher amount of dsRNA template available for processing into viral siRNAs by Dicer-2.

For *Ae*. *japonicus*, viral 21 nt siRNAs were most abundant, yet viral piRNAs with characteristic ping-pong signatures were not identified, thus suggesting that siRNA-mediated activity is the major RNAi pathway in *Ae*. *japonicus*. Likewise, 21 nt viral-derived siRNAs were the major group of small RNAs present in ZIKV-infected *Ae*. *aegypti* and USUV-infected *Cx*. *pipiens* but no piRNAs were detected in these mosquito species [9,37]. In our study, we identified a major hotspot of 21 nt small RNAs at the 3’ UTR of the positive viral RNA strand of ZIKV. This peak, present at the 3’ SL, was only observed for ZIKV-infected *Ae*. *japonicus* mosquitoes but not for USUV-infected *Ae*. *japonicus* mosquitoes. The position of the peak and the length of the small RNA are identical to a previously described microRNA-like small RNA which is produced from the 3’ SL of the 3’ UTR of WNV [38]. This viral small RNA, named KUN-miR-1, is known to facilitate virus replication in mosquito cells [38]. Moreover, a similar viral small RNA hotspot has also been found during WNV infection in *Cx*. *pipiens* mosquitoes [39]. However, an earlier study with a different *Cx*. *pipiens* colony did not detect this hotspot in USUV-infected mosquitoes [9]. This viral small RNA hotspot at the 3’ SL of the 3’ UTR thus only seems to be present for certain combinations of flaviviruses and mosquito species.

Interestingly, we found AejapNV1 in our *Ae*. *japonicus* mosquitoes. This narnavirus is most closely related to Zhejiang mosquito virus 3 detected in mosquitoes collected in Australia [40] (sequence identity: 78%) and China [41] (sequence identity: 77%). Classical members of the *Narnavirus* genus only code for an RdRp on the positive-sense viral RNA strand, and are historically known to infect yeasts and oomycetes [42]. However, we found strong 21 nt siRNA responses against AejapNV1 whereas the most abundant small RNAs produced by yeasts and oomycetes are primarily 22–26 nt in length [43,44], which strongly suggests that AejapNV1 replicates in *Ae*. *japonicus*. Additionally, AejapNV1 contains a second ORF on the negative-sense viral RNA strand, which encodes a protein of no known homology. This ambisense coding strategy has also been found for multiple other narnaviruses discovered in mosquitoes [40,41] and for a narnavirus detected in mosquito *Culex tarsalis* CT cells [24], which could suggest the existence of a novel group of narnaviruses containing a second ORF that enables replication in its insect host. The effect of narnavirus persistent infections on the vector competence of mosquitoes for arboviral diseases such as ZIKV and USUV is currently unknown and remains to be investigated. Further studies on for example the tissue tropism of AejapNV1 could provide insights into the possible interference of this virus with pathogenic flaviviruses.

In Europe, *Cx*. *pipiens* is considered the main vector for USUV [8]. Out of the *Cx*. *pipiens* mosquitoes that ingested a blood meal containing USUV, 69% was reported to experimentally transmit USUV [9], which is considerably higher than the percentage of blood fed *Ae*. *japonicus* mosquitoes that transmitted USUV in our study. Given that *Cx*. *pipiens* is a competent vector for USUV and that this mosquito is abundantly present in Europe, it likely plays a more important role in USUV outbreaks in Europe than *Ae*. *japonicus*. In addition, USUV-positive *Ae*. *albopictus* mosquitoes have also been detected in Europe [8], however the exact role of this invasive mosquito in USUV transmission needs to be studied in more detail.

ZIKV transmission by the primary vector *Ae*. *aegypti* and the secondary vector *Ae*. *albopictus* was found to be more efficient than ZIKV transmission by *Ae*. *japonicus* in our study [28,45]. For *Ae*. *aegypti* Rockefeller, only a minor mosquito salivary gland barrier is restricting ZIKV transmission whereas a midgut barrier was found to be absent [28]. In our study, we found a strong midgut barrier restricting virus dissemination in *Ae*. *japonicus*, suggesting that there is an intrinsic difference in the midgut of *Ae*. *aegypti* and *Ae*. *japonicus*, which strongly affects the vector competence of these mosquitoes for ZIKV. Importantly, the high prevalence of *Ae*. *albopictus* in Mediterranean Europe [46] received recent attention due to the recognition of three locally acquired human ZIKV cases in the south of France during the summer of 2019, which were likely caused by human-to-human transmission via *Ae*. *albopictus* mosquitoes [47–49]. The fact that *Ae*. *albopictus* seems to be a competent vector for ZIKV in France, underlines the need to study in more detail whether *Ae*. *japonicus* could also initiate ZIKV transmission in the Netherlands. In general, arbovirus outbreaks are notoriously hard to predict and not all parameters contributing to the efficiency of a certain mosquito population to transmit an arbovirus are always known in sufficient detail. The efficiency of a vector to transmit a pathogen in the field is often referred to as the vectorial capacity. Besides vector competence, the vectorial capacity of a mosquito species also depends on mosquito population density, mosquito survival, host preference, feeding frequency and behaviour of the mosquito, and environmental conditions [50]. A thorough understanding of all these contributing factors is needed to assess the risk of transmission of arboviruses such as ZIKV and USUV by *Ae*. *japonicus* in the field.

Infectious droplet feeding has proven to be an efficient method to provide a blood meal to field-collected *Ae*. *japonicus* mosquitoes that do not feed on the conventional Hemotek feeding system. Inside a vertebrate host or the artificial Hemotek, the blood with virus is usually kept at a temperature ranging from 37°C to 40°C, whereas during infectious droplet feeding, the blood droplets with virus are kept at room temperature. The temperature difference between these ways of infectious blood feeding could potentially have an effect on virus entry in the mosquito midgut cells. Currently, there is no indication that infectious droplet feeding at room temperature positively or negatively influences the vector competence of mosquitoes for ZIKV. Infection and transmission rates of *Ae*. *aegypti* mosquitoes in our study were similar as reported in previous experiments where *Ae*. *aegypti* mosquitoes were provided with an infectious blood meal using a Hemotek feeding system [28]. Another caveat of our study is that artificial feeding on infectious blood droplets might not reflect feeding on infectious hosts under natural conditions. Artificial feeding of *Ae*. *aegypti* mosquitoes with ZIKV resulted in lower transmission potential compared to *Ae*. *aegypti* mosquitoes blood fed on viraemic mice [51]. Moreover, it has been suggested that ZIKV transmission by mosquitoes is enhanced after engorgement of a second blood meal [52]. *Ae*. *japonicus* could therefore be a more competent vector for ZIKV and USUV when feeding under natural conditions in the field instead of receiving an artificial infectious blood meal by droplet feeding. Thus, it remains challenging to determine the real risk of ZIKV and USUV transmission by *Ae*. *japonicus*.

Environmental conditions such as temperature are important determinants of arbovirus transmission by mosquitoes [53,54]. In this study, we found transmission of ZIKV by *Ae*. *japonicus* at 28°C. Moreover, we also found replicating ZIKV in the bodies of *Ae*. *japonicus* after oral exposure of these mosquitoes to ZIKV and subsequent incubation at 25°C. This indicates that ZIKV can also infect *Ae*. *japonicus* at temperatures lower than 28°C, which supports previous findings where after incubation as low as 21°C, the bodies of *Ae*. *japonicus* mosquitoes tested ZIKV-positive [19]. In the same study, ZIKV dissemination but not transmission was reported for mosquitoes kept at 24°C [19]. Follow-up studies with high numbers of mosquitoes at temperatures lower than 28°C could give more insight into the true risk of ZIKV and USUV transmission by *Ae*. *japonicus* in north-western European countries such as the Netherlands, as average summer temperatures in these areas are usually around 18°C [55].

At the moment, we do not have strong indications that *Ae*. *japonicus* represents a major risk for public health in Europe, as widespread *Ae*. *japonicus* populations are currently present in e.g. Germany and Austria, and to the best of our knowledge, no arboviral outbreaks have so far been linked to the presence of *Ae*. *japonicus* in these areas. Nevertheless, we found that *Ae*. *japonicus* can experimentally transmit ZIKV and USUV at 28°C. This implies that in the case of large, expanding populations of *Ae*. *japonicus* and high environmental temperatures, this mosquito could potentially be involved in future arboviral outbreaks. Thus, we need to consider *Ae*. *japonicus* as a potential vector for ZIKV and USUV in Europe.
